# Changes in the Ileal, but Not Fecal, Microbiome in Response to Increased Dietary Protein Level and Enterotoxigenic *Escherichia coli* Exposure in Pigs

**DOI:** 10.1128/AEM.01252-19

**Published:** 2019-09-17

**Authors:** Jolinda Pollock, Michael R. Hutchings, Kate E. K. Hutchings, David L. Gally, Jos G. M. Houdijk

**Affiliations:** a Animal and Veterinary Sciences, Scotland’s Rural College (SRUC), Edinburgh, United Kingdom; b The Roslin Institute and Royal (Dick) School of Veterinary Studies, University of Edinburgh, Edinburgh, United Kingdom; The Pennsylvania State University

**Keywords:** ETEC, gut, ileum, microbiome, nutrition, pigs, protein

## Abstract

The relationship between porcine gut microbiota composition and health is an important area of research, especially due to the need to find alternatives to antimicrobial use to manage disease in livestock production systems. Previous work has indicated that lower crude dietary protein levels can reduce the impacts of postweaning colibacillosis, which is a porcine diarrheal disease caused by enterotoxigenic Escherichia coli (ETEC). Here, to explore the complex interactions between the gut microbiota, protein nutrition, and ETEC exposure, the microbial compositions of both ileal digesta and feces were analyzed with or without ETEC exposure from pigs fed a low- or high-protein diet. Since ETEC colonization is mostly localized to the ileum, changes in the small intestinal microbiota were expected in response to ETEC exposure. This was supported by the study findings, which identified significant microbiota changes in ileal samples but not in fecal samples. Both increased dietary protein and ETEC exposure impacted on ileal microbiota alpha diversity (richness and diversity indices) and beta diversity (structure, stability, and relative taxon abundances) at certain sampling points, although the combination of a high-protein diet and ETEC exposure had the most profound impact on ileal microbiota composition. An understanding of how infection and nutrition lead to microbiota changes is likely to be required if dietary strategies are to be developed for the management of enteric diseases.

**IMPORTANCE** Gut bacterial communities have been shown to play a key role in pig health and development and are strongly influenced by host diet, but studies highlighting the complex interactions between nutrition, gut infections and the microbiome tend to focus on bacterial populations in the feces and not other important gut locations. We found that alteration of dietary protein level and exposure to a pathogenic microorganism, enterotoxigenic Escherichia coli (ETEC), changed bacterial populations in the distal small intestine (i.e., the ileum). We found that the most profound changes occurred in pigs fed a high-protein diet in combination with exposure to ETEC, showing a clear interaction between dietary composition and exposure to a key pathogen. These changes were not observed in the fecal samples, revealing the importance of studying biologically pertinent sites in the gut, and so the data will help to inform the development of alternative management strategies for enteric disorders.

## Abstract (precis)

Gut bacterial communities have been shown to play a key role in pig health and development and are strongly influenced by host diet, but studies highlighting the complex interactions between nutrition, gut infections and the microbiome tend to focus on bacterial populations in the feces and not other important gut locations. We found that alteration of dietary protein level and exposure to a pathogenic microorganism, enterotoxigenic Escherichia coli (ETEC), changed bacterial populations in the distal small intestine (i.e., the ileum). We found that the most profound changes occurred in pigs fed a high-protein diet in combination with exposure to ETEC, showing a clear interaction between dietary composition and exposure to a key pathogen. These changes were not observed in the fecal samples, revealing the importance of studying biologically pertinent sites in the gut, and so the data will help to inform the development of alternative management strategies for enteric disorders.

## INTRODUCTION

Due to concerns about the overuse of antimicrobial agents leading to multidrug resistance, attention is now being focused on more restrictive use of these drugs in food-producing animals (1, 2). Despite the ban which was put in place on antibiotics for growth promotion in the European Union in 2006, antimicrobial agents are still being used extensively in the swine industry to prevent and treat enteric infections (3). One such disease is postweaning colibacillosis (PWC), which is an economically important intestinal disease (4, 5) that has been variably linked to a decreased growth rate under clinical or subclinical conditions (6–8). PWC is most commonly caused by enterotoxigenic Escherichia coli (ETEC), with ETEC O149:K91:F4 strains being one of the most dominant serotypes affiliated with PWC worldwide (9), which primarily colonize the ileal mucosa in the small intestine (10). Antimicrobial resistance has been demonstrated in E. coli strains isolated from pigs with PWC from a variety of locations (11–15).

To overcome the economic losses associated with such diseases while avoiding the overuse of antimicrobial agents, the development of alternative management strategies needs to be considered (16). Dietary manipulation as a control measure for PWC has been considered due to previous evidence that reducing crude protein (CP) levels can lower disease severity (17, 18). Specifically, while amino acid supply from dietary protein is essential for a range of bodily functions, including maintenance, growth (protein deposition), and immune responses, when provided in surplus to digestive capacity, bacterial fermentation of excess protein and the resultant production of irritant, carcinogenic, and potentially toxic by-products such as ammonia, indole, cresol, and skatole can occur (19, 20). As such, excess protein has been identified as a key risk factor for the development of PWC (21), and indeed lowering dietary protein content has been shown to reduce fecal scores (i.e., reduce diarrhea incidence [22]) and to decrease ETEC counts in the ileal digesta (23) and in feces (24).

Disruption of the gut microbiota during the weaning transition has been cited as a key influence leading to the emergence of PWC (25), and the alteration of dietary protein levels has been shown to cause shifts in the *Lactobacillus*/coliform ratio using culturing methods (21, 22) and in particular bacterial taxa by terminal restriction fragment length polymorphism (23). The complex interactions between dietary protein and the gut microbiota have more recently been explored using 16S rRNA gene metabarcoding, with differing dietary protein levels (26) and sources (27) being shown to influence the ileal microbiota in finisher and weaner pigs, respectively. However, there are no published studies that explore the interactions between dietary protein level, ETEC exposure, and gut microbiota composition using 16S rRNA gene metabarcoding. The aim of this study was to test the hypotheses that (i) increased dietary protein, (ii) ETEC exposure, and (iii) their interaction affect ileal and fecal microbiota composition.

## RESULTS

### Weight gain, feed intake, and fecal consistency scores.

There were no significant treatment effects on the average daily weight gain (ADG) over the entirety of the trial (*P* > 0.05) considering pigs that were subject to postmortem on day 13. When considering data from all pigs included in the trial, there were again no significant effects of ETEC exposure or dietary protein level on the ADG (*P* > 0.05) (Table 1). However, feeding treatment did affect the average daily feed intake (ADFI) between days 5 and 9, with feed intake being around 10% greater in pigs on a low-protein (LP) diet than in pigs on a high-protein (HP) diet (*P* = 0.047) (Table 2).

**TABLE 1:** Average daily weight gain over the specified time periods in the four experimental groups

Period (days)	ADG (g/pig/day) for the following inoculation group:				SEM	Probability (*P*) for the following parameter:		
	SHAM LP	ETEC LP	SHAM HP	ETEC HP		Diet	Exposure	Interaction
0–2	−40		6		35	0.193		
2–5	303	277	250	285	36	0.384	0.857	0.238
5–9	337	385	365	350	36	0.888	0.528	0.225
9–13	383	490	449	431	45	0.926	0.170	0.058

**TABLE 2:** Average daily feed intake over the specified time periods in the four experimental groups

Period (days)	ADFI (g/pig/day) for the following inoculation group:				SEM	Probability (*P*) for the following parameter:		
	SHAM LP	ETEC LP	SHAM HP	ETEC HP		Diet^*a*^	Exposure	Interaction
0–2	63		84		13	0.129		
2–5	278	277	269	294	20	0.786	0.416	0.357
5–9	428	404	404	391	25	0.047*	0.960	0.463
9–13	586	674	574	569	46	0.080	0.209	0.159

a *, statistically significant (*P* < 0.05).

Prior to ETEC exposure (i.e., days 1 and 2), the mean fecal consistency scores across all treatment groups ranged between 1 and 1.06, highlighting that the feces were generally well formed. There was no effect of ETEC exposure on fecal consistency score across the entire experiment (*P* = 0.67). However, pigs fed the HP diet had significantly higher fecal consistency scores over the course of the trial (HP = 1.19 ± 0.09, LP = 1.11 ± 0.07; *P* = 0.03).

### ETEC quantification.

**(i) Ileal digesta samples.** At baseline (i.e., day −1), all digesta samples obtained tested negative for the presence of the *faeG* gene. In addition, throughout the experiment, all SHAM (i.e., sham-exposed pigs using 3 ml of sterile phosphate-buffered saline [PBS]) LP and SHAM HP pigs tested were negative for the presence of ETEC F4 (i.e., ETEC O149:K91:F4 [STa/STb/LT/EAST1]).

**FIG 1:**
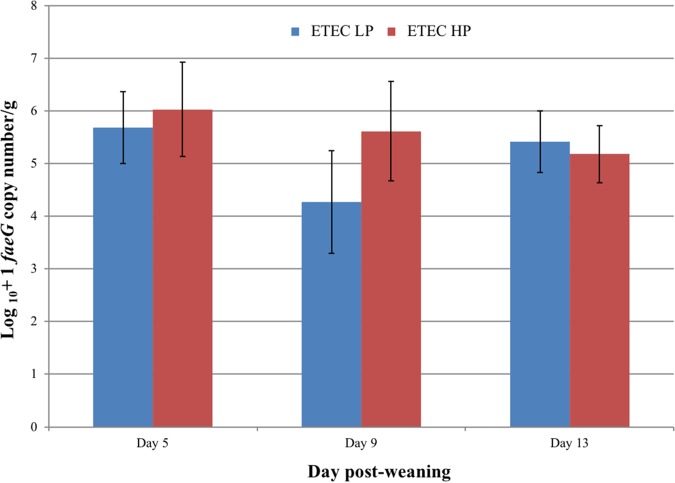
Mean log_10_+1 *faeG* gene copy number (± the standard error of the mean [SEM]) per gram of wet ileal digesta at each postmortem point. On day −1, *faeG* was not detected in any samples from both ETEC LP and ETEC HP groups.

On days 5 and 9, ETEC-exposed pigs fed the HP diet showed a higher mean ETEC load in the ileal contents compared to those fed the LP diet (Fig. 1). On day 13, the mean levels of ETEC F4 in ileal digesta from ETEC LP pigs increased compared to day 9 and a small reduction was observed in the mean ETEC load in ETEC HP pigs. Although the mean number of *faeG* copies was higher in ETEC HP pigs on day 5 and 9 in comparison to ETEC LP pigs, there were no statistically significant differences at any of the postmortem points (*P* > 0.05).

**(ii) Fecal samples.** All fecal samples obtained at baseline tested negative for the presence of the *faeG* gene. In addition, all fecal samples obtained from pigs in the SHAM LP and SHAM HP treatment groups tested negative for ETEC F4 throughout.

**FIG 2:**
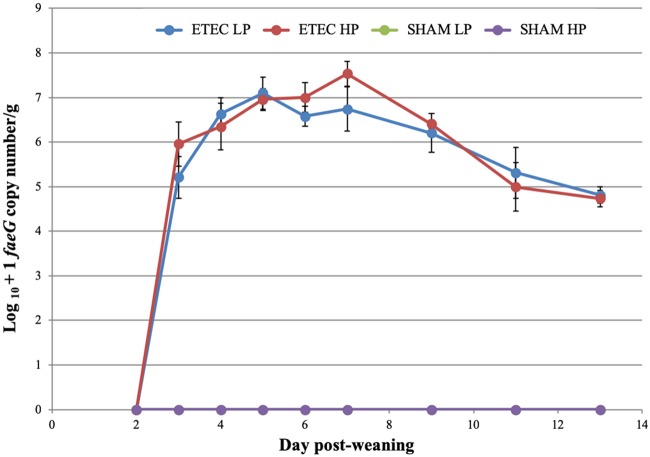
Mean log_10_+1 *faeG* gene copy number (± the SEM) per gram of wet feces prior to ETEC exposure (day 2) and after exposure (days 3 to 7, 9, 11, and 13).

The mean log_10_+1 *faeG* copy number/g wet feces values obtained from ETEC LP and ETEC HP pigs subjected to postmortem analyses on day 13 are presented in Fig. 2. On day 3 (1 day postexposure), ETEC HP pigs were shedding higher numbers of ETEC than were ETEC LP pigs. On day 7 (5 days postexposure) the greatest difference between the ETEC LP and ETEC HP treatment groups was observed, with the ETEC HP pigs shedding almost 10-fold more ETEC F4 than the ETEC LP pigs. From days 7 to 13, both treatment groups showed a steady decrease in mean ETEC counts. However, there were no statistically significant effects of the dietary protein level on the ETEC shedding level in the feces over the trial duration (*P* = 0.24).

### Quality control of sequence data.

Poor quality sequences and sequencing artifacts from both ileal and fecal samples amounted to 15% of the sequences and were discarded. On average, 102,674 ± 55,780 reads were analyzed per sample, and 681 bacterial phylotypes were identified. To ensure that sequencing depth was adequate for this experiment, Good’s coverage was calculated. All samples had a Good’s coverage of >0.99, highlighting that an estimated 99% of the bacteria contained in both ileal digesta and fecal samples were captured during sequencing. Using the mock bacterial community data, the sequencing error rate in both Illumina MiSeq runs was calculated to be 0.01%. In the reagent-only controls, low DNA yields were evident according to the spectrophotometer measurements (NanoDrop 1000; Thermo Scientific, United Kingdom), with background DNA contamination detected postsequencing. These sequences were phylogenetically diverse with low read numbers within each phylotype.

### Effects of ETEC exposure on gut microbiota: alpha and beta diversities of fecal and ileal communities.

When considering fecal samples, there were no significant effects of ETEC exposure on bacterial richness or diversity at any time point (Table 3). In addition, there were no significant differences in community structure or stability on day 5, 9, or 13 when the ETEC and SHAM groups were compared. There were also no significant differences in the expression of relative abundances at any time point when comparing the ETEC and SHAM groups (*P* > 0.05).

**TABLE 3:** Impact of dietary protein and ETEC exposure on gut microbial community richness (Chao 1) and diversity (ISI) in feces at selected days postweaning

Day and analysis	Mean Chao 1 and ISI values ± SD for the following inoculation group:				Probability (*P*) for the following parameter:		
	SHAM LP	SHAM HP	ETEC LP	ETEC HP	Exposure	Dietary protein	Interaction
5							
Chao 1	74.52 ± 9.12	78.84 ± 6.63	74.90 ± 9.57	76.62 ± 6.13	>0.05	>0.05	>0.05
ISI	10.26 ± 2.07	11.38 ± 3.40	9.89 ± 2.90	10.90 ± 1.96	>0.05	>0.05	>0.05
							
9							
Chao 1	75.41 ± 5.74	77.00 ± 10.87	67.66 ± 7.31	76.86 ± 11.35	>0.05	>0.05	>0.05
ISI	9.58 ± 2.22	9.99 ± 4.36	8.51 ± 2.52	11.05 ± 2.05	>0.05	>0.05	>0.05
							
13							
Chao 1	73.67 ± 6.56	74.96 ± 7.23	73.28 ± 11.05	71.03 ± 12.85	>0.05	>0.05	>0.05
ISI	11.12 ± 3.35	11.37 ± 3.60	9.75 ± 3.43	10.25 ± 4.43	>0.05	>0.05	>0.05

When examining the ileal digesta samples, there were no significant effects of ETEC exposure on either bacterial community richness or diversity on day 5 or 13 (*P* > 0.05). However, on day 9, significant effects of ETEC exposure on both community richness and diversity were evident, with the ETEC group having higher ileal microbiota richness and diversity indices (Table 4).

**TABLE 4:** Impact of dietary protein and ETEC exposure on gut microbial community richness (Chao 1) and diversity (ISI) in ileal digesta at selected days postweaning

Day and analysis	Mean Chao 1 and ISI values ± SD for the following inoculation group:				Probability (*P*)^*a*^ for the following parameter:		
	SHAM LP	SHAM HP	ETEC LP	ETEC HP	Exposure	Dietary protein	Interaction
5							
Chao 1	67.56 ± 28.81	82.89 ± 45.88	68.30 ± 40.63	48.40 ± 44.33	>0.05	>0.05	>0.05
ISI	2.76 ± 1.26	4.86 ± 4.39	3.03 ± 2.92	2.77 ± 1.61	>0.05	>0.05	>0.05
							
9							
Chao 1	36.01 ± 20.67	44.26 ± 18.36	45.10 ± 16.42	80.08 ± 25.83	0.03*	0.03*	0.04*
ISI	1.66 ± 0.54	2.45 ± 0.82	2.59 ± 0.72	5.85 ± 2.81	0.03*	0.003*	>0.05
							
13							
Chao 1	32.24 ± 11.69	46.52 ± 18.97	40.50 ± 29.57	53.64 ± 14.24	>0.05	0.02*	>0.05
ISI	2.79 ± 1.01	3.72 ± 1.92	3.18 ± 1.90	3.96 ± 2.31	>0.05	>0.05	>0.05

a *, statistically significant (*P* < 0.05).

On days 5 and 9 (i.e., 3 days and 7 days postexposure, respectively), there was no impact of ETEC exposure on ileal microbiota structure (*P* > 0.05). However, on day 13 (i.e., 11 days postexposure), the ETEC and SHAM groups had significantly different ileal microbiota structures (*P* = 0.024). In addition, pigs exposed to ETEC had a significantly less stable ileal microbiota on day 5 (*P* = 0.03) and day 13 (*P* = 0.04) than the SHAM pigs. There were no associated phylotype-level changes in relative abundances at any of the sampling points when comparing ileal communities in the ETEC and SHAM groups (*P* > 0.05).

### Effects of dietary protein level on gut microbiota: alpha and beta diversities of fecal and ileal communities.

There were no significant effects of dietary protein level on fecal microbiota richness or diversity at any time point (*P* > 0.05) (Table 3). There were also no effects of dietary treatment on fecal microbiota structure (*P* > 0.05) or stability (*P* > 0.05). Likewise, there were also no differentially expressed phylotype relative abundances when fecal samples obtained from LP and HP pigs were compared (*P* > 0.05).

In ileal digesta samples, on day 5, there were no significant differences in community richness or diversity between LP and HP pigs (Table 4). However, on day 9, significant differences in both richness and diversity were found when LP and HP pigs were compared, with the HP pigs having higher mean ileal community richness and diversity indices versus the LP group. On day 13, there were no significant differences in diversity, but significant differences in richness were still evident, with the HP pigs continuing to show a higher mean Chao 1 index.

There were no effects of dietary treatment on ileal microbiota structure or stability on day 9 (*P* < 0.05). However, there were significant differences in both community structures and stabilities on days 5 and 13 between LP and HP groups (*P* < 0.05). These changes in community structure were associated with changes in relative abundances of particular bacterial phylotypes, though on day 13 only. HP pigs had greater abundances of the order *Burkholderiales*, *Lactobacillus* spp., and Lactobacillus salivarius than LP pigs (*P* < 0.05) and smaller abundances of Campylobacter fetus (*P* = 0.027).

### Interactive effects of ETEC exposure and dietary protein level on gut microbiota.

When considering fecal samples, there were no interactive effects of dietary protein level and ETEC exposure on community richness or diversity at any time point (*P* > 0.05) (Table 3). There were also no interactive effects of ETEC exposure and dietary protein level on fecal microbiota structure or stabilities at any time point (*P* > 0.05).

When considering ileal digesta samples, there were no interactive effects of treatment on microbial community richness or diversity on days 5 and 13 (*P* > 0.05). However, on day 9, there were significant interactive effects of ETEC exposure and dietary treatment on community diversity (*P* = 0.04), with the ETEC HP group showing the highest mean Inverse Simpson index (ISI) and the SHAM LP group having the lowest (Table 4).

When we compared the ileal microbial communities from samples obtained from pigs in all four treatment groups, we observed significant differences in community structure (*P* = 0.017) and stability (*P* = 0.04) on day 13 only. There were no differences in community structure or stability when comparing the SHAM LP and SHAM HP groups at any time point (*P* > 0.05). However, there were differences in both community structure and stabilities when the ETEC LP and ETEC HP groups were compared on both days 5 and 13, with the ETEC HP group having less-stable ileal communities at both time points (*P* < 0.05).

## DISCUSSION

The aim of this experiment was to assess the sensitivity of ileal and fecal bacterial communities to ETEC exposure and/or increased dietary protein level. Since the majority of gut microbiota studies focus on fecal microbial communities, the novel approach of this work was to study microbiota dynamics at a key site of ETEC colonization (i.e., the ileum) where the effects of these factors may be more likely to be detected.

### Nutrition, ETEC exposure, and pig health and performance.

There were no significant effects of ETEC exposure on pig health showing that the model, as intended, produced a subclinical disease state with absence of diarrhea and minimal and transient effects on feed intake and weight gain, which has been shown previously (8, 23, 24). Although ETEC exposure did not have measurable pathology or consistent effects on performance and health, the bacteria clearly colonized the pigs, since ETEC was excreted in the feces for the full duration of the experiment following the single exposure on day 2, and it was thus still relevant to study the impact of ETEC exposure and interactions with dietary protein level on fecal and ileal microbiota composition.

We did observe that feed intake was around 10% higher in the pigs fed the LP diet specifically between days 5 and 9, though the achieved CP intake was still 18% greater for HP pigs than for LP pigs. Since body weight gain was not affected, the former observation supports the view that pigs may overcome the relatively small degree of dietary protein scarcity through their feeding behavior. Similarly, weaned pigs fed 23 and 18% CP rations grew at the same rate, but those fed 13% CP rations failed to increase intake and thus overcome protein scarcity, as their weight gain was compromised (22). Although there was sufficient variation in dietary protein level to induce a consistent effect on fecal score, a larger variation may have been required to have a more profound impact on health and performance (24). ETEC exposure combined with the HP diet caused statistically significant increases in fecal score which has been shown previously (28). Although statistically significant, we do not believe that this difference is biologically important, as all feces were well formed throughout the experiment.

### The fecal microbiota did not change in response to ETEC exposure and dietary treatments.

In this study, the fecal microbiota was not significantly altered by ETEC exposure. In previous work, exposure to ETEC has been shown to have an impact on fecal bacterial populations, specifically with the observation of a decreased *Lactobacillus*/coliform ratio, with dietary formulations similar to those described here (29). Conversely, in more recent work by our group using 16S rRNA gene metabarcoding, no significant effects of ETEC exposure were observed on bacterial community composition in feces (30). A key site of colonization for ETEC is the small intestine, so any effects exerted may be localized to this gut section (30). In agreement, less-profound effects of Salmonella enterica serovar Typhimurium challenge were found on fecal microbial communities compared to ileal samples from weaner pigs (31), highlighting the importance of studying relevant gut sections compared to excreted feces and the challenge of obtaining fecal microbial signatures from biological events upstream in the gut.

Fecal microbial populations were also not significantly impacted by an increased dietary protein level. Other researchers have found that a moderate protein level change did not shift fecal microbiota populations as dramatically as in small intestinal samples (26) or did not influence them at all (32). Conversely, previous work has shown feces-level alterations in microbial composition in response to changes in the dietary protein level (33) in grower pigs. Taking the findings of this study and previous work into account, although conflicting, it is important to consider that the fecal microbiota may not be the most suitable target for exploring the impact of experimental treatments.

### The ileal microbiota changed in response to ETEC exposure.

Even in the absence of effects on host health and performance measures, statistically significant changes in ileal bacterial populations were linked to ETEC exposure. This occurred in the presence of large within-group variations, which has been noted in previous human (34) and pig (35) studies. ETEC was consistently detected in the ileal digesta samples throughout the duration of the experiment, showing persistence in the small intestine after a single inoculation, which is consistent with a pilot study informing this protocol (36).

ETEC-exposed pigs had less-stable ileal microbial communities, meaning that there was more microbial variation within this treatment group in comparison to the control group. Alpha diversity indices were greater in the ileal samples from the ETEC groups at 7 days postinoculation than in the SHAM groups, with differences in ileal microbiota structure and stability also evident at 11 days postinoculation. We speculate that ETEC exposure destabilized more dominant members of the ileal microbiota, which consequently may have caused a surge of rarer microbial taxa. The decrease in ileal microbiota stability in the ETEC group suggests that exposure has likely variably affected each individual’s ileal microbiota. Profound changes in ileal microbial communities have previously been shown in young pigs in the presence of ETEC (37), porcine epidemic diarrhea virus (38), and *Salmonella* challenge (31), with the former occurring in the absence of clinical disease, a finding reflected in the present study.

### The ileal microbiota changed in response to increased dietary protein.

Increasing the level of dietary protein in the weaner pig diet significantly increased ileal bacterial community richness and diversity on day 9, which has been found in previous work in finisher pigs (26). This suggests that lowering dietary protein may have inhibited the growth of specific intestinal bacteria, such as protein-fermenting bacteria (39).

Ileal microbial populations were structurally different on days 5 and 13 postweaning when comparing pigs fed low- and high-protein diets. In other work, the ileal microbiota structure in gilts was found to be significantly different in pigs fed 14, 16, and 17% CP diets (40). In the present study, on day 13 only, this change in structure was associated with differences in specific phylotype relative abundances. This suggests that these taxa do not respond immediately to increased dietary protein but shift in abundance in response to prolonged exposure in HP conditions. Namely, pigs fed the high-protein diet showed higher levels of the order *Burkholderiales* and *Lactobacillus* in the ileal digesta on day 13, with the ileal (40) and cecal (41) dominance of *Lactobacillus* in pigs fed an HP diet also shown in previous work. Some members of the *Lactobacillus* genus have proteolytic properties (42) and are consequently likely to have increased in response to greater protein availability. Microbial utilization of amino acids has been shown to start in the distal small intestine (43, 44) and, consequently, the increased dietary protein is likely to have influenced ileal populations in the present study.

### Interactive effects of dietary protein level and ETEC exposure.

There were more pronounced variations in ileal microbiota structure when we compared HP and LP diets for the ETEC groups than for the SHAM groups, with the ETEC HP group also having the least-stable ileal communities (3 and 11 days postinoculation) and the highest alpha diversity indices (7 days postinoculation). This suggests that the combination of ETEC exposure and the HP diet used here caused the most profound ileal community shifts. These population shifts may be partly explained by the ETEC HP group having higher mean levels of ETEC in the small intestine than did the ETEC LP group, with 10-fold-higher numbers of ETEC being detected from samples obtained from these pigs at 5 days postinoculation. It is known that ETEC colonization is complex and multifactorial, with elevated dietary protein level facilitating this action (45), which may in part explain the higher ETEC numbers isolated from the ETEC HP pigs.

### Conclusion.

In conclusion, increased dietary protein levels and ETEC exposure significantly changed ileal microbial communities, with their combination resulting in the most pronounced changes in ileal bacterial populations. This study has highlighted the importance of considering the spatial variation in the microbiota, particularly as in this case, the ileum is crucially a key site of ETEC colonization in the porcine gut. These findings also have implications for the development of nutritional management strategies for PWC, particularly when aiming to manipulate the host gut microbiota.

## MATERIALS AND METHODS

### Pigs and housing.

The animal experiment described was reviewed and approved by Scotland's Rural College's (SRUC's) Animal Welfare and Ethical Review Body and carried out under Home Office regulations (PPL 60/4489).

Pigs (Large White × Landrace) were weaned at 25.0 ± 0.8 days of age (means ± the standard deviation) and weighed 9.10 ± 1.27 kg at weaning (day 0). Pigs were housed in groups of four in 4-m^2^-square pens, which were cleaned and bedded with fresh sawdust daily. A single feeder and nipple drinker were included, with both water and feed being provided *ad libitum* for the trial duration. The environmental temperature was set at 25°C for the first 4 days and was decreased by 2°C for the remainder of the experiment. The shed lights were switched on between 8:00 and 18:00 and night lights were maintained between 18:00 and 8:00.

### Feeding treatments.

Prior to weaning, all piglets had access to a standard creep feed (digestible energy [DE] = 16.3 MJ/kg, CP = 230 g/kg) from around day −7 to weaning. Thereafter, pigs were offered one of two experimental diets, formulated to provide CP at 187 (LP) or 244 (HP) g/kg. The compositions of the experimental diets are listed in Table S1 in the supplemental material, and the diets were modeled to reflect the diet ingredients previously used (29). The elevated level of CP was achieved by increasing inclusion levels of the main animal and plant protein sources (fishmeal, soya protein concentrate, dried skimmed milk powder, and dried full-fat whey powder) by 54%, largely at the expense of micronized wheat. Diets were formulated to be balanced for DE, lactose, and essential amino acid/lysine ratios as much as possible through additional changes in oil, lactose, and synthetic amino acid inclusion levels, respectively. Feed was sampled during daily feed weighing and was pooled per treatment for proximate analysis and assessment of amino acid composition (see Table S1). The determined CP levels for LP and HP were 180.9 and 228.8 g/kg, respectively, which were slightly lower in level and smaller in difference than formulated.

### ETEC exposure treatments.

An ETEC O149:K91:F4 (STa/STb/LT/EAST1) strain (ETEC F4) isolated from a pig diagnosed with clinical PWC (SRUC Veterinary Services, United Kingdom) was revived by streaking it onto a MacConkey agar plate, followed by incubation at 37°C for 24 h. A well-isolated bacterial colony was immersed in 5 ml of brain heart infusion broth containing nalidixic acid (15 μg/ml) and incubated for 24 h at 37°C (with shaking) to produce a stationary-phase culture. Bacterial cells were then harvested via centrifugation, and the pellet was washed three times in 25 ml of sterile phosphate-buffered saline (PBS). The pellet was resuspended, and an inoculum containing an estimated 10^8^ CFU/ml was prepared. The optical density at 600 nm of the inoculum was measured using a spectrophotometer (Ultrospec 2100 Pro; Fisher Scientific, United Kingdom) to estimate the bacterial cell concentration. For a more accurate *post hoc* confirmation of ETEC counts, the inoculum was serially diluted in PBS and enumerated on MacConkey agar plates in triplicate.

On day 2 (i.e., 2 days postweaning), pigs were either orally administered 3 ml of the ETEC inoculum by syringe (ETEC) or, for sham-exposed pigs, 3 ml of sterile PBS in the same manner (SHAM). This dose was trickled slowly at the back of the mouth to ensure that a swallowing reflex took place. A pilot experiment showed that this protocol allows for ETEC establishment, as evidenced by prolonged fecal ETEC excretion and the presence of ETEC on ileal tissue as the predilection site for at least 6 days postdosing (36).

### Experimental design.

This experiment was carried out over four experimental rounds between 9 July and 14 October 2015. A total of 144 pigs across 16 litters were included in the trial (4 litters per round), with pens being balanced for weaning weight, sex, and litter origin within each round. The experiment consisted of four treatments arranged in a 2 × 2 factorial combination of the feeding treatments (LP versus HP) and ETEC exposure treatments (ETEC versus SHAM).

After baseline samples were obtained on day −1 (i.e., 1 day preweaning), i.e., one pig from each litter/round combination (*n* = 16), the remaining pigs were assigned to one of the four experimental treatment combinations (*n* = 32 pens), i.e., ETEC LP, ETEC HP, SHAM LP, and SHAM HP, with four pigs being assigned to each pen at day 0. A serial-slaughter design was implemented whereby pigs were removed for postmortem on days 5, 9, and 13 as described in Table S2. At each postmortem point, pigs were selected from each pen to maintain balance across experimental treatments for weaning weight, sex, and litter origin.

### Weight gain, feed intake, and health.

Pigs were weighed on days −1 (preexposure baseline pigs only), 0, 2, 5, 9, and 13 postweaning. The average daily feed intake (ADFI) per pig was estimated daily by subtracting the weight of feed refused from the weight of feed offered the preceding day, divided by the number of pigs in the pen during that day, while average daily weight gain (ADG) per pig was calculated for the periods 0 to 2, 2 to 5, 5 to 9, and 5 to 13 days postweaning. Pigs that were subject to postmortem on day 13 were used to assess the impact of the experimental treatments on ADG over the trial duration.

Fecal consistency, cleanliness, and overall health scores were recorded daily on a pen basis, using a subjective four-point scale described previously (22). Briefly, for fecal consistency scoring, an increase in score represents an increase in fecal fluidity (i.e., 1, normal; 2, normal diarrhea; 3, watery diarrhea; and 4, dysentery). Increases in both cleanliness and overall health scores represent an increase in fecal contamination and a deterioration of health, respectively. Throughout the experiment, all pigs remained in good health, which was subjectively observed as actively responding to human presence and all pigs having clean pink skin, bright eyes, and upright ears.

### Postmortem sampling and DNA extractions.

Pigs were sedated using a mixture of medetomidine (0.01 ml/kg at 1 mg/ml), midazolam (0.1 ml/kg at 5 mg/ml), ketamine (0.1 ml/kg at 100 mg/ml), and azaperone (0.025 ml/kg at 40 mg/ml). Pigs were then euthanized by intracardiac injection of pentobarbital (0.7 ml/kg at 200 mg/ml). The abdomen was opened from the pubis to the sternum to reveal the gastrointestinal tract. The cecum was isolated and tied off at the ileal-cecal junction before measuring 10 cm cranially and tying off again with string. The ileal digesta was emptied into a universal tube before being snap-frozen on dry ice. A fecal sample was also taken directly from the rectum at postmortem and was also snap-frozen on dry ice.

All samples were stored at –80°C for a maximum of 2 weeks prior to DNA extraction, which was carried out using a MoBio PowerSoil DNA isolation kit (now branded as the DNeasy PowerSoil kit; Qiagen, United Kingdom), as described previously (30).

### Temporal fecal sampling and DNA extractions.

ETEC-exposed pigs that underwent postmortem on day 13 were subject to fecal screening for ETEC F4 over several time points to monitor individual shedding levels throughout the trial (*n* = 32). Two sham-exposed pigs from each round were also sampled and screened to verify that SHAM pigs did not shed ETEC F4, and this selection was balanced for treatment across the four experimental rounds (*n* = 8).

Fecal samples were taken directly from the rectum daily between days 2 and 7 and on days 9, 11, and 13. These samples were immediately snap-frozen on dry ice prior to storage at –80°C. DNA extractions were carried out using a MoBio PowerSoil DNA isolation kit, again as previously described (30).

### ETEC F4 enumeration.

Fecal and ileal levels of ETEC F4 were measured using quantitative PCR (qPCR), which targeted the *faeG* major fimbrial subunit. Reactions were set up using Brilliant III Ultra-Fast SYBR green qPCR master mix (Agilent Technologies, USA) and the primers F4-463F (5′-GGTTCTGAACTCTCGGCTGG-3′) and F4-597R (5′-AGAACCTGCGACGTCAACAA-3′), as developed and described previously (30). The values obtained were used to estimate the number of ETEC F4 bacteria by establishing the number of gene copies/g of wet ileal digesta or feces.

### 16S rRNA gene metabarcoding.

All ileal digesta and fecal samples obtained at postmortem were subjected to 16S rRNA gene metabarcoding targeting the V3 hypervariable region, as described previously (30). DNA concentrations of the purified libraries were then measured using a Qubit 3.0 fluorometer (Thermo Fisher Scientific, United Kingdom) using a Qubit double-stranded DNA high-sensitivity assay kit (Thermo Fisher Scientific). Using the readings obtained by the Qubit instrument, four library pools were constructed using equimolar concentrations of DNA from each sample. A reagent-only control and mock bacterial community (HM-782D; BEI Resources, ATCC, Manassas, VA) were included as part of each sequencing run to assess background contamination, sequencing error rate, and inter-run variability. On submission to the sequencing center (Edinburgh Genomics, United Kingdom), library pools were quantified using a Quant-iT PicoGreen double-stranded DNA assay kit (Thermo Fisher Scientific) to ensure a sufficient yield for sequencing. Sequencing was carried out using an Illumina MiSeq platform (Illumina, USA), using V2 chemistry and producing 250-bp paired-end reads. The generated sequences (with primers removed) are available publicly through the European Nucleotide Archive (ENA) under accession number PRJEB33396.

### Sequence processing and analysis.

The generated sequences were subjected to processing and quality control using the mothur software package (version 1.36.0) (46), as detailed previously (30). A total of 35,540,010 contiguous sequences were generated; 15% of these sequences that contained ambiguous bases, that were of incorrect length, or that contained homopolymers of >8 bp were removed. A further 5% of these sequences that did not map to the reference alignment or were not identified as bacterial sequences were also removed. The remaining sequences were clustered into phylotypes based on their similarity to reference sequences and were subsampled (*n* = 5,000) for analysis.

The Inverse Simpson index (ISI) was calculated for each sample to measure alpha diversity, and the Chao 1 index was calculated to assess richness. To test whether there were significant differences in diversity and richness between treatments, analysis of variance (ANOVA) was carried out using Genstat 16 (VSN International, United Kingdom). Distance matrices were compiled by using Yue and Clayton theta similarity coefficients (47), which take into account both community membership and relative abundance. Two distance matrices were created: one for all fecal samples and one for all ileal digesta samples. The statistical significance of any clustering by time point or treatment was assessed by analysis of molecular variance (48). The statistical significance of variation between populations was assessed using homogeneity of molecular variance (49). To identify bacterial phylotypes that were significantly different between treatment groups, Metastats (50) was used, and the *P* values were corrected using the false discovery rate. The subsampled data set was simplified to only include phylotypes which were ≥0.1% of the relative abundance at each time point examined.

### Statistical analysis of ADG, ADFI, fecal consistency scores, and qPCR data.

Statistical analyses were carried out using Genstat 16 (VSN International, United Kingdom) unless stated otherwise. ADFI data were assessed using repeated measures analysis of variance (RM-ANOVA) to establish any main or interactive temporal effects of ETEC exposure and dietary protein level. These analyses included ETEC exposure status and dietary protein level as main factors and experimental round as a block. The ADG for each experimental period, as well as over the entire experiment, was assessed using ANOVA to establish whether there were any main or interactive effects of ETEC exposure and dietary protein level.

To assess the uniformity over time of the fecal consistency scores and whether there were any main effects of ETEC exposure or dietary protein level, an ordinal logistic regression was performed using Minitab 17 (Minitab, Inc., USA). The categorical indicators (i.e., fecal consistency scores) were assigned as the response, and time point, ETEC exposure status, and dietary protein level were assigned as categorical predictors. To assess whether there were statistically significant effects of dietary protein level on both ileal ETEC load and fecal ETEC shedding, ANOVA and RM-ANOVA were carried out, respectively, with dietary protein level being included as a main factor and experimental round as a block, using data from the ETEC-exposed pigs only.

### Accession number(s).

Sequence data are available publicly through the European Nucleotide Archive (ENA) under accession number PRJEB33396.

## Supplementary Material

Supplemental file 1
