# Supplementary material for: Changes in the Ileal, but Not Fecal, Microbiome in Response to Increased Dietary Protein Level and Enterotoxigenic Escherichia coli Exposure in Pigs
Source: Appl Environ Microbiol. 2019 Sep 17;85(19):e01252-19. doi: 10.1128/AEM.01252-19 (PMC6752020; doi:10.1128/AEM.01252-19)
Supplement: Supplemental file 1 [file AEM.01252-19-s0001.pdf]

Table S1: Ingredients, chemical composition and calculated digestible energy of the two experimental diets.

Ingredients (g/kg)	Low Protein	High Protein
**Micronized wheat**	314.9	237.2
**Cooked dehulled oats**	125.0	125.0
**Micronized maize**	125.0	125.0
**Fishmeal**	32.5	50.0
**Full fat soya**	20.0	20.0
**Dried-skim milk powder**	91.0	140.0
**Soya protein concentrates**	96.7	149.0
**Fat-filled whey**	32.5	50.0
**Vegetable oil**	34.4	10.1
**Lactose**	101.0	74.2
**Limestone**	0.8	1.5
**Dicalcium phosphate**	14.7	7.8
**Salt**	1.9	0.3
**l-Lysine-HCl**	1.6	1.1

**dl-Methionine**	1.4	1.8
**l-Threonine**	1.2	1.5
**l-Tryptophan**	0.2	0.3
**Premix**	5.2	5.2
**Crude protein**	180.9	228.8
**Lysine**	10.3	13.2
**Methionine**	5.7	6.0
**Threonine**	8.3	9.9
**Tryptophan**	1.7	2.6
**Ash**	47.7	46.1
**Acid hydrolysed ether extract**	51.9	53.8
**Neutral detergent fibre**	69.1	59.8
**Digestible energy (MJ/kg)[a]**	16.0	16.0

[a] Calculated from in-house matrix at feed mill.

Table S2: The number of pigs included in each of the experimental groups. The post-mortem day (day post-weaning), the number of pigs per treatment and the appropriate protein level/exposure treatment combination for each treatment (low protein (LP) and high protein (HP), ETEC-exposed (+) and sham-exposed (-)) are described.

Treatment ID	PM day(s)	Number of pigs	CP level	ETEC exposure
**Baseline**	-1	16	N/A	N/A
**5-ETEC LP**	5	8	LP	+
**5-ETEC HP**	5	8	HP	+
**5-SHAM LP**	5	8	LP	-
**5-SHAM HP**	5	8	HP	-
**9-ETEC LP**	9	8	LP	+
**9-ETEC HP**	9	8	HP	+
**9-SHAM LP**	9	8	LP	-
**9-SHAM HP**	9	8	HP	-
**13-ETEC LP**	13	16	LP	+
**13-ETEC HP**	13	16	HP	+
**13-SHAM LP**	13	16	LP	-
**13-SHAM HP**	13	16	HP	-
